# Scientific journal publishing is too complex to be measured by a single metric: time to review the role of the impact factor!

**DOI:** 10.1590/0074-02760160005

**Published:** 2016-09

**Authors:** Claude Pirmez, Adeilton Alves Brandão, Hooman Momen

How should scientific authors choose the best journal to disseminate their research work? If this question was formulated two centuries ago by a biomedical researcher in Europe, the answer would be quite simple: decide first in which language the message should be delivered (*e.g.*, English, German, French, Russian, Latin, Spanish) and then pick one out of the approximately 100 science journals which in that period were publishing papers in one of those languages. The world of scholarly publishing at the beginning of nineteenth century was so small (https://conscicom.org/) that scientists could quickly decide to which journal to send the manuscript.

Times have changed, and in the second decade of the twenty first century, choosing which journal to publish a manuscript in is not only a question of which expert audience a scientist wants to reach, but also and paramountly, it is a matter of professional survival and prestige. Publish or Perish, would say those in the most competitive organisations, or else, Publish or Paris, would say those in a good mood. In such a difficult environment, the overwhelming majority of scientists would first ask: “what is the impact factor (IF) of this journal?”. Nothing appears to be more definitive than the simple metric based on the two years arithmetic mean of citations for papers in that journal. IF has become the answer of our times for measuring the quality of science: a single metric that has maximum priority over all the other qualifications of a scientific journal.

The scientific world today is saturated by almost 30,000 journals delivering more than 3,000,000 articles a year in all fields of knowledge (http://www.scimagojr.com). Do we really need such an avalanche of scientific information (or should we say, scientific waste)? This is a corollary of the problem on measuring the quality of scientific journals and their published papers. An interesting point of view on the problem between quality and quantity in scientific publishing is discussed by Sarewitz (2016). Whether publishing more or less articles would improve the quality of scientific work is matter of debate, but certainly less articles entering the submission systems of journals would save precious time and resources for both editors and reviewers.

The misuse (or the correct use!) of the IF and related metrics has long been debated by the academic community, editors and funding agencies [see, for example, an algorithm proposed by NIH researchers as an alternative to evaluate grant proposals (Hutchins et al. 2016)]. Some scientific societies have also launched alerts on the indiscriminate use of the impact factor, culminating in the DORA (San Francisco declaration on research assessment) (http://www.ascb.org/dora/), and Leiden manifesto (Hicks et al. 2015). But has the IF been created with such a goal and scope? In the words of its creator, Eugene Garfield, the results of the IF would “*...appear to be of great potential value in the management of library journal collections. Measures of citation frequency and impact factor should be helpful in determining the optimum makeup of both special and general collections...*” (Garfield 1972).

Things can get more complicated when this metric is deployed to measure and define what is beyond its natural limits. This is the case for the use of the IF as a proxy to indicate the quality and the productivity of individual research output. Such a function is clearly not within the scope of the IF metric, which originally was supposed to allow librarians to recommend the subscription of journals with the best “benefit-cost ratio” to their institutional decision makers (Garfield 1972).

Considering all the stakeholders with an interest in scientific publishing (*e.g.*, authors, reviewers, readers, academics, editors, librarians, funding bodies, research institutions, for profit and non profit organisations), it is beyond doubt that no single metric can provide a common objective for all stakeholders. A journal is only a vehicle to convey the scientific information targeted to a specific audience. A high IF in general indicates a high quality journal; however, this quality is not automatically transferred to every article published in a general. Articles should be judged on their individual merits and not on the journal of their publication. Other metrics such as citations and altmetric scores are better for measuring the quality and impact of individual articles. Perhaps valorizing the article level metrics is the most important change we have to introduce in the scientific publishing arena.

*Claude Pirmez* Editor in chief *Adeilton Alves Brandão* *Hooman Momen* Editors
